# MiR-493-5p inhibits Th9 cell differentiation in allergic asthma by targeting FOXO1

**DOI:** 10.1186/s12931-022-02207-2

**Published:** 2022-10-17

**Authors:** Xingyu Rao, Heting Dong, Weili Zhang, Huiming Sun, Wenjing Gu, Xinxing Zhang, Li Huang, Yongdong Yan, Chuangli Hao, Wei Ji, Canhong Zhu, Zhengrong Chen

**Affiliations:** 1 Department of Respiratory Medicine, Children’s Hospital of Soochow University, Jingde Road No. 303, Suzhou, 215003 Jiangsu China; 2 First Affiliated Hospital of Gannan Medical University, Ganzhou, 341000 China

**Keywords:** Asthma, FOXO1, miR-493-5p, Th9 cell differentiation, Exosomes

## Abstract

The role of micro RNAs (miRNAs) in asthma remains unclear. In this study, we examined the role of miRNA in targeting FOXO1 in asthma. Results showed that miR-493-5p was one of the differentially expressed miRNAs in the PBMCs of asthmatic children, and was also associated with Th cell differentiation. The miR-493-5p expression decreased significantly in the OVA-induced asthma mice than the control groups. The miR-493-5p mimic inhibited the expression of the IL-9, IRF4 and FOXO1, while the inhibitor restored these effects. Moreover, the Dual-Luciferase analysis results showed FOXO1 as a novel valid target of miR-493-5p. According to the rescue experiment, miR-493-5p inhibited Th9 cell differentiation by targeting FOXO1. Then the exosomes in association with the pathogenesis of asthma was identified. Various inflammatory cells implicated in asthmatic processes including B and T lymphocytes, DCs, mast cells, and epithelial cells can release exosomes. Our results demonstrated that the DC-derived exosomes can inhibit Th9 cell differentiation through miR-493-5p, thus DC-derived exosomal miR-493-5p/FOXO1/Th9 may serve as a potential therapeutic target in the development of asthma.

**Supplementary Information:**

The online version contains supplementary material available at 10.1186/s12931-022-02207-2.

## Introduction

Bronchial asthma (abbreviated as asthma) is one of the children’s most common and severe chronic airway inflammatory disease. In recent years, the incidence and mortality of asthma have gradually increased worldwide, posing a great threat to children’s health and aggravating financial stress on families and society. Asthma has become a global public health issue and raised great concerns. Developing intervention measures from the pathogenesis level of asthma is the key treatment, which is complicated and hard to identify.

Current studies on pathogenesis of asthma is still unclear. Most researchers considered the inhibition of helper T cell type 1 (Th1) and the hyperfunction of helper T cell type 2 (Th2) caused Th1/Th2 imbalance as the main reason. But Th1/Th2 imbalance cannot explain all the experimental phenomena of asthma because it may involve other cell populations. With the discovery of Th9 cells, a new subgroup of CD4^+^Th cells, more studies focused on the role of Th9 cells in the immunopathology of asthma showed that these cells can be differentiated from the Naïve CD4^+^T cells stimulated by IL-4 and TGF-β, producing a large amount of IL-9 [1], which is reported that Th9/IL-9 is pathogenic in asthma [2, 3]. Moreover, a significant changes of several cytokines related to Th9 cell differentiation in mouse asthma model was detected compared with the control mice group, including forkhead box O1(FOXO1), IL-4, IL-9, IRF4, Spi1, BATF, and IRF1 [2, 4], (Additional file 1: Fig. S1). Therefore, inhibition of Th9 cells differentiation might be a potential immunotherapy for asthma.

The miRNAs are well-known short non-coding RNAs that regulate gene expression by complementary base pairing with the 3′-untranslated region (3′UTR) of mRNAs [5]. There is an increasing trend of studies focused on miRNAs because of their functional effect on polarization of CD4^+^T cells. Through high-throughput sequencing, we found that there were many abnormal miRNAs changes in PBMCs of asthmatic children compared with non-asthmatic ones. Further gene ontology (GO) analysis and Kyoto Encyclopedia of Genes and Genomes (KEGG) signaling pathway analysis showed that a significant downregulated PBMCs of asthmatic children was related to T cell differentiation. In addition, the transcription factor FOXO1 was reported to be a positive regulator of Th9 cell differentiation by binding to promoters of IL-9 and IRF4 [6]. Therefore, FOXO1 was considered as a potential target gene of miR-493-5p from TargetScan.

Researchers also studied the pathogenesis of asthma in association with exosomes [12]. Exosomes are small vesicles (30–100 nm) that enable cellular communication by shuttling different molecules such as nucleic acids, lipids, and proteins, and have been considered as efficient cell-to-cell messengers that can cross biological barriers and modulate the immune system [7–11]. Various inflammatory cells implicated in asthmatic processes including B and T lymphocytes, DCs, mast cells, and epithelial cells can release exosomes [12]. Exosomes from DCs contain molecules and will stimulate T-cell responses [13]. Therefore, we investigated the effect of miR-493-5p on the role of Th9 cell differentiation by targeting FOXO1. Our results proved that DC-derived exosomal miR-493-5p could inhibit Th9 cell differentiation by targeting FOXO1.

## Materials and methods

### Children’s asthma data

Patients diagnosed with asthma according to the guidelines of the Global Initiative for Asthma [14] were enrolled from the department of pulmonary medicine at the Children’s Hospital of Soochow University. The non-atopic healthy subjects were included as a control group. Peripheral blood samples from 5 patients with asthma exacerbation and 5 healthy children were used for high throughput sequencing. All the subjects were instructed to avoid the use of asthma medication (such as glucocorticoids) 4 weeks before testing. This study was approved by the local Ethics Committee of the Children’s Hospital of Soochow University. All participants had signed the informed consent.

### Mice asthma experiment

To develop the OVA-induced asthma model, 6–8-week-old female mice BALB/c (weight 20 ± 2 g) were divided into four groups according to the experimental demand (n = 8). 2 mg OVA (Sigma, SLBP0782V) was dissolved with 4 ml PBS containing 40 mg aluminum hydroxide (Sinopharm), and 100 μl of the mixture was injected into the abdominal cavity of the experimental mice on day 0 and day 7. Following that, the mice were treated with inhalational OVA (5%) for 30 min per day from day 17 to day 20 to construct the asthma model. Three days before challenge, 50 μl (10 nmol) of miR-493-5p agomiR or 50 μl agomiR NC were delivered by nasal drop for 3 days. Mice in the control groups were treated with PBS. The animal experiment has been approved by the Ethics Committee of Suzhou University (No. SUDA20210512A01). The testing process followed the 3R principle (Reduction, Replacement, Refinement).

### Measurement of airway hyperreactivity

Mice were anesthetized for the measurement of pulmonary mechanics 24 h after the last OVA challenge. Mice were anesthetized with 50 mg/kg pentobarbital and instrumented for the measurement of pulmonary mechanics (BUXCO Electronics), then were tracheostomized, intubated, and mechanically ventilated at a tidal volume of 0.2 ml and a frequency of 150 breath/min. Lung resistance (RL) was measured in response to increasing doses (3 to 20 mg/ml) of aerosolized acetyl-β-methylcholine chloride methacholine (Sigma-Aldrich).

### Lung specimens of mice

After the measurement of airway hyperreactivity, mice were euthanasia by dislocation of the cervical spine, then lace the mouse on a dissection surface with the ventral side facing up. Wipe the entire ventral surface of mouse with sterile alcohol pads saturated with 70% isopropanol to disinfect. Mount the mouse with all four legs restrained. The trachea was exposed and 0.5 ml PBS was used for 3 consecutive lavages to obtain BALF. Gently open the thoracic cavity by slowly incising the rib cage through the mediastinum, the left lung tissues were fixed in 10% neutral-buffered formalin (48 h) and embedded in paraffin fixation. Then, the paraffin-embedded sections were stained with hematoxylin and eosin (H&E), periodic acid-Schiff (PAS) to evaluate the lung inflammatory. The remaining lung tissues were analyzed by flow cytometry, Western Blot, enzyme-linked immuno sorbent assay (ELISA) and quantitative polymerase chain reaction (qPCR).

### Mouse lung cells isolation

The perfused lungs were transferred into a sterile and pyrogen-free 60 mm culture dish with 3 ml PBS. Lung tissue were minced with a scalpel to < 1 mm size. Then 300 µg/ml Liberase TL and 5U/ml DNase I were added, mixed by pipetting and incubated at 37 ℃ in an incubator for 25 min. Gently mixed once by pipetting after 10 min, then passed the dissociated lungs through a 100 µm cell strainer installed on a 50 ml conical tube. The back of a plunger from 1 ml syringe was used to mash up cell clumps on the filter. The filter was washed with 20 ml wash buffer (PBS + 2%Heat inactivated FBS + 2 mM EDTA),then centrifuged at 500×*g* at 4 ℃ for 5 min, discarded the supernatant and resuspended the cell pellet in 20 ml wash buffer, repeated these steps and repaired single-cell suspension by adding 200 μl PBS to the cell pellet.

### Induction of Th9 cells

PBMC cells were obtained by density gradient centrifugation from the peripheral blood of widetype mice, and the CD4^+^T cells were separated using Biotin anti-mouse CD4 (Biolegend, 100508) and Streptavidin MicroBeads (Miltenyi Biotec, 130-048-101) according to the instructions. Then CD4^+^T cells was incubated with Th9 induction medium [5 µg/ml anti-CD3, 5 µg/ml anti-CD28, 10 ng/ml IL-4 (PeproTech) and 10 ng/ml TGF-β (GenScript)] for 7 days, and the medium was replaced every three day. The cells were transfected with miR-493-5p mimic/inhibitor under the condition of inducing Th9 cell differentiation. Consequently, cells were collected to detect IL-9 by Flow cytometry, FOXO1, IL-9, IRF4 mRNA and protein levels were detected by qPCR, ELISA and Western blot, respectively.

### BMDC-derived exosome isolation and co-culture with CD4^+^T cells

BMDCs were generated from BM using a conventional method [15]. BMDCs were cultured at 1 × 10^6^ cells/ml in RPMI 1640 (Gibco, USA), then stimulated with IL-33(15 ng/ml) and TSLP(15 ng/ml). The culture medium supernatants were collected to isolate exosomes using ultracentrifugation by the Optima L-100XP Ultracentrifuge (Beckman Coulter, USA) [16]. 1 × 10^6^cells/ml CD4^+^T cells were incubated in with 60 ug of BMDC-derived exosomes in Th9 condition, and IL-9 were detected by flow cytometry. Then exosomes were isolated from peripheral blood of asthma mice and control mice, qPCRwas used to determine the serum exosomal miR-493-5p. At last, BMDC-derived exosome-miR-493-5p NC and exosome-miR-493-5p inhibitor were constructed and incubed with Naïve T cells in Th9 condition for 72 h respectively, and IL-9 were detected by flow cytometry.

### Flow cytometry

Cells were adjusted to 1 × 10^6^ cells/ml and cultivated with 2 μl Monensin for 6 h, and then washed with 1 × perm/wash buffer and centrifuged. 100 μl of staging buffer and 0.5 μl of CD4 antibody (Biolegend) was mixed into each tube, and then incubated in a refrigerator at 4 ℃ in dark for 30 min. Cells were washed twice with 1 × perm/wash buffer, and 250 μl fixation/permeabilization solution (BD GolgiStopTM) was injected into each tube, then the cells were incubated in refrigerator at 4 ℃ for 20 min. The cells were washed with 1 × perm/wash buffer and then centrifugated, 0.5 μl of IL-9 antibody (BioLegend) was mixed with 100 μl of Staining Buffer, then added into each tube. Incubated in a refrigerator at 4 ℃ in dark for 30 min, the cells were washed twice with 1 × perm/wash buffer, and then resuspended with Staining Buffer. The quantitative detection was performed immediately by flow cytometry (BD-FACSVerse). Data were analyzed with FlowJo software (FlowJo, Ashland, OR).

### RNA extraction and RT-qPCRanalysis

Total RNA was extracted from experiment cells using Trizol (Invitrogen, Carlsbas, USA). RNA was revere-transcribed into cDNA using PrimeScript TM RT reagent kit (Takara, Japan). Reverse transcript qPCR Master Mix was purchased from Takara, Japan. The primer sequences are shown in Table 1.

**Table 1:** Primer sequences used for RT-qPCR

Gene	Species	Type	Sequence
FOXO1	Mouse	Forward	GGGTCCCACAGCAACGATG
		Reverse	CACCAGGGAATGCACGTCC
IRF4	Mouse	Forward	CTTTGAGGAATTGGTCGAGAGG
		Reverse	GAGAGCCATAAGGTGCTGTCA
IL-9	Mouse	Forward	ACACCGTGCTACAGGGAGG
		Reverse	TGGTTGCATGGCTTTTCG
IRF1	Mouse	Forward	CTCAGCAGCTCTACCCTACC
		Reverse	CACTCAGAGAGACTGCTGCT
Spi1	Mouse	Forward	TCAGCCAAGCCAGATCAGAA
		Reverse	AGAGGAGCTGACATTGGCAT
BATF	Mouse	Forward	GAAGAGCCGACAGAGACAGA
		Reverse	TCCTCGGTGAGCTGTTTGAT
miR-493-5p	Mouse	Forward	GCCGAGTTGTACATGGTAGG
		Reverse	CTCAACTGGTGTCGTGGA
U6	Mouse	Forward	CTCGCTTCGGCAGCACA
		Reverse	AACGCTTCACGAATTTGCGT
β-actin	Mouse	Forward	GTCCCTCACCCTCCCAAAAG
		Reverse	GCTGCCTCAACACCTCAACCC

### ELISA

All samples were preserved at − 80 ℃ for subsequent assay of IL-9 by ELISA (Elabscience, E-EL-M0724c) according to the instructions. The concentration was calculated according to the corresponding OD value.

### Western blot analysis

The tissues or cells were collected and lysed using radio immunoprecipitation assay buffer (Beyotime Institute of Biotechnology, China) supplemented with PMSF (Beyotime Institute of Biotechnology). Equivalent protein quantities were subjected to SDS-PAGE and transferred to polyvinylidene fluoride membrane. Then, the blots were blocked with 5% fat free milk at room temperature and incubated overnight at 4℃ with the primary antibodies: anti-IL-9 (ab203386; abcom), anti-FOXO1 (AF603; Beyotime), anti-IRF4 (646402-1; biolegent), and anti-β-actin mouse mAb (AP0060; Bioworld). Blots were then washed and incubated with the appropriate secondary antibodies [goat anti-rabbit or goat anti-mouse (Bioworld)] for 1 h. Membranes were visualized with BeyoECL Plus (Beyotime).

### Small interfering FOXO1 transfection

Three siRNAs targeting FOXO1 were conducted. In brief, siRNA were added into six-well plates with CD4^+^T cells and then treated by using Lipofectamine2000 (Invitrogen) according to the operation manual. After 48 h, CD4^+^T cells were collected for further experiments. Then, qPCR analysis was applied to detect FOXO1 expression and to validate the transfected efficiencies. According to the interfering efficiencies, siFOXO1-1 was selected for the next experiment.

### Dual-luciferase assay

The FOXO1 3′UTR sequence containing miR-493-5p binding site was synthesized and inserted into psiCHECK2 luciferase vector plasmid to establish FOXO1 3′UTR wild-type (WT) plasmid. On this basis, point mutation kit was used to mutate the binding site of miR-493-5p on FOXO1-WT plasmid, thus establishing FOXO1 3′UTR mutant (MUT) plasmid. The FOXO1-WT and FOXO1-Mut fragments were subcloned into the firefly luciferase gene psiCHECK2 reporter vectors (Promega, USA) to generated psiCHECK2-FOXO1 3′UTR WT and psiCHECK2-FOXO1 3′UTR MUT vectors, respectively. After that, the above vectors were co-transfected with miR-493-5p mimic or negative control into 293T cells for 6 h. Finally, firefly and renilla luciferase activities were sequentially measured using dual-luciferase assays (Promega) 48 h after the transfection and evaluated by the Bio Tek™Microplate Reader.

### Statistical analysis

Statistical analyses were performed by using SPSS software (version 24). All experiments were repeated at least three times independently and data were presented as the mean ± standard deviation. Differences between two experimental groups were analyzed by student’s t-test, and differences among more than two groups were evaluated by one-way analysis of variance (ANOVA). *P* value < 0.05 was considered statistically significant.

## Results

### miRNA in PBMCs of asthmatic children

**Fig. 1:**
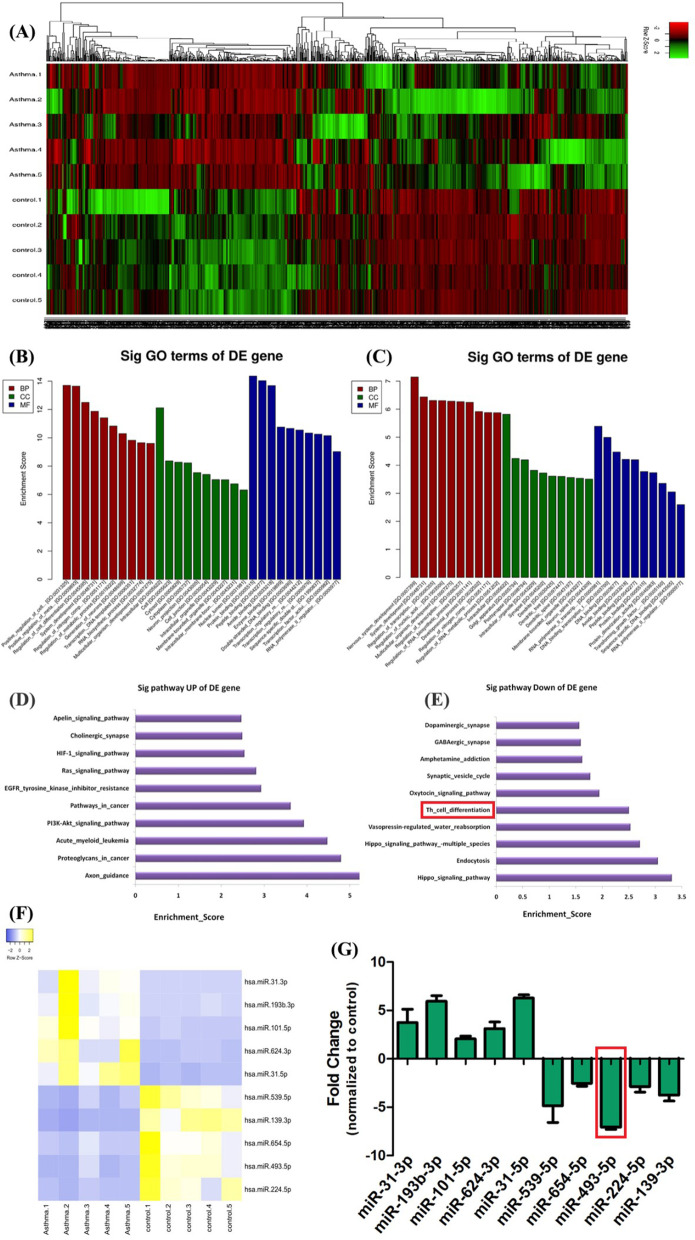
High throughput sequencing PBMCs of asthmatic children and 10 miRNAs related to Th cell differentiation. Peripheral blood was collected from asthmatic children (n = 5) and non-asthmatic children (n = 5), and PBMCs were isolated. High throughput sequencing was used to screen different expression of miRNAs. There were 506 miRNAs with fold change > 1.5 and P < 0.05, among which 268 up regulated and 238 down regulated (**A**). GO analysis showed that these aberrant dysregulated miRNAs were related to DNA binding, transcription factor activity, cell inflammation, cell activation, proliferation and so on (**B**). KEGG signal pathway analysis showed that these miRNAs were closely related to Th cell differentiation, and their expression was down regulated in asthmatic children (**C**). Through informatics analysis and published database, 10 miRNAs (5 up-regulated and 5 down-regulated) involved in Th cell differentiation were selected (**D**–**F**). Among them, miR-493-5p was selected due to large differences between groups and small differences within groups (**G**)

Peripheral blood from which PBMCs isolated were collected from asthmatic children, and high throughput sequencing was used to screen different expression of miRNAs. There were 506 miRNAs with fold change > 1.5 and *P* < 0.05, among which 268 upregulated and 238 downregulated (Fig. 1A). GO analysis showed that these aberrant dysregulated miRNAs were related to DNA binding, transcription factor activity, cell inflammation, cell activation, proliferation and so on (Fig. 1B). KEGG signal pathway analysis showed that these miRNAs were closely related to Th cell differentiation, and their expression was downregulated in asthmatic children (Fig. 1C).

According to informatics analysis and published database, 10 miRNAs (5 up-regulated and 5 down-regulated) involved in Th cell differentiation were selected for further study (Fig. 1D–F). The large differences between groups and small differences within groups from the miR-493-5p (Fig. 1G) suggesting it may critical to Th cell differentiation in asthmatic children.

### miR-493-5p and Th9 cells differentiation

**Fig. 2:**
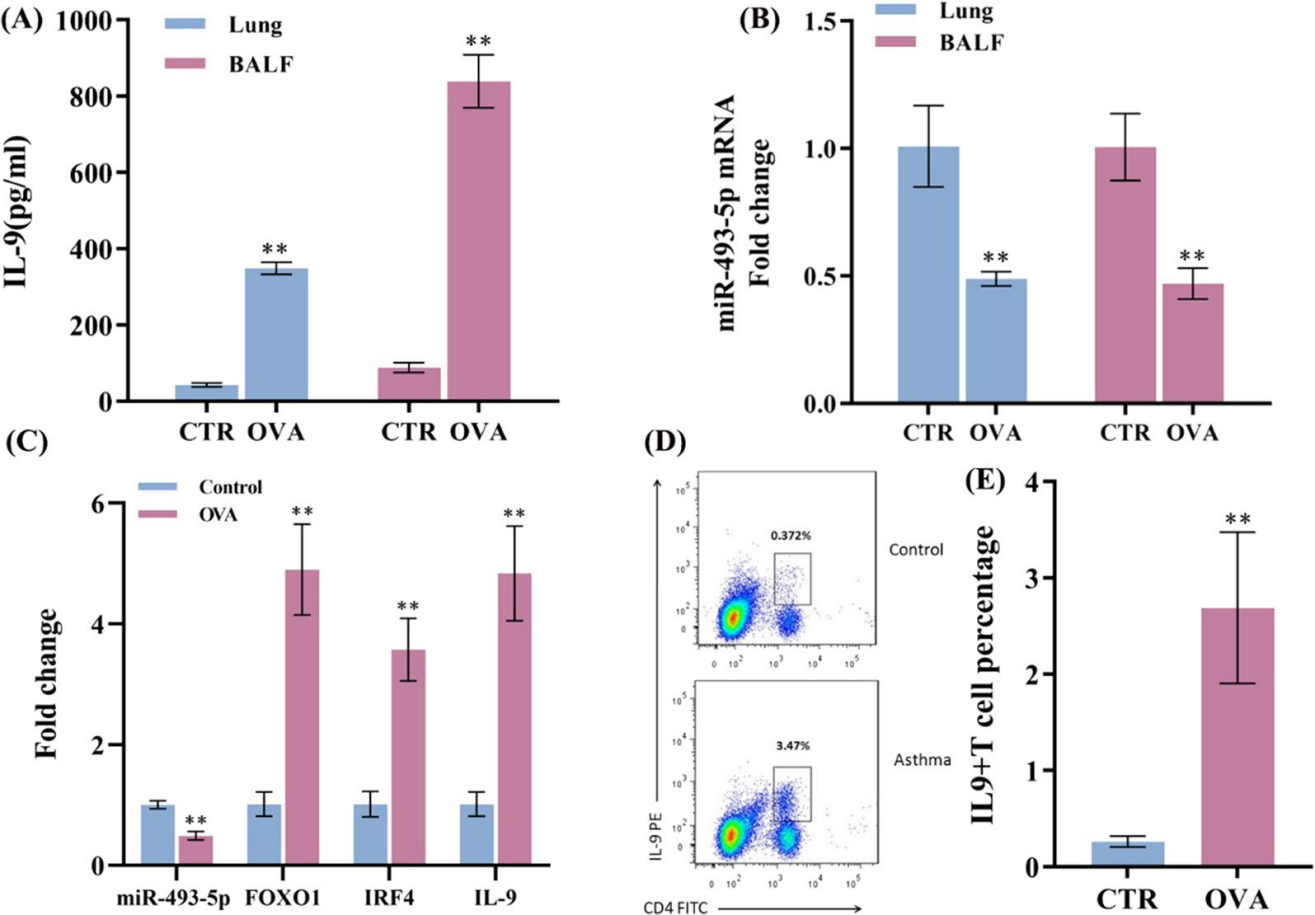
Down regulation of miR-493-5p and higher proportion of Th9 cells in murine asthma models. OVA was used to induce allergic airway inflammation in mice. The mice were euthanasia by dislocation of the cervical spine after the measurement of airway hyperreactivity, and then their lung tissues and BALF were collected (n = 8 for each group). **A** The IL-9 levels in lung tissue and BALF were measured by ELISA, and IL-9 level in lung tissue and BALF of asthma groups were significantly higher than the control groups. **B** The miR-493-5p expression in lung tissue and BALF were detected by RT-PCR, and the miR-493-5p expression were both obviously diminished in asthma groups compared with control groups. **C** MiR-493-5p, FOXO1, IRF4 and IL-9mRNA expression in lung tissues were detected by RT-PCR, and IL-9, FOXO1, IRF4 expression were all significantly upregulated in lung tissues from asthmatic mice. **D**, **E** The proportion of CD4^+^T cells secreting IL-9 in PBMCs were analysed by flow cytometry, the results revealed that the higher proportion of CD4^+^T cells secreting IL-9 in the PBMCs of the asthma mice. ^**^P < 0.01, compared to control group

The OVA was used to induce allergic airway inflammation in mice. The mice were euthanasia after the measurement of airway hyperreactivity, and then the lung tissues and BALF were collected for the following study. As shown in Fig. 2A, IL-9 level in lung tissue and BALF of asthma groups (n = 8 for each group) were significantly higher than the control groups (n = 8 for each group). Compared with control groups, the miR-493-5p expression were both obviously diminished in asthma groups (Fig. 2B). The marker cytokine of Th9 cells is IL-9, and FOXO1, IRF4 were reported to be transcription factors that promoted Th9 cell differentiation. As the RT-qPCR data showed in Fig. 2C, IL-9, FOXO1 and IRF4 expression were all significantly upregulated in lung tissues from asthmatic mice. Meanwhile, the Flow cytometry results revealed the higher proportion of CD4^+^T cells secreting IL-9 in the PBMCsof the asthma mice (Fig. 2D and E). These data suggested that them iR-493-5p was down regulated, but Th9 cell proportion was increased in asthma mice.

**Fig. 3:**
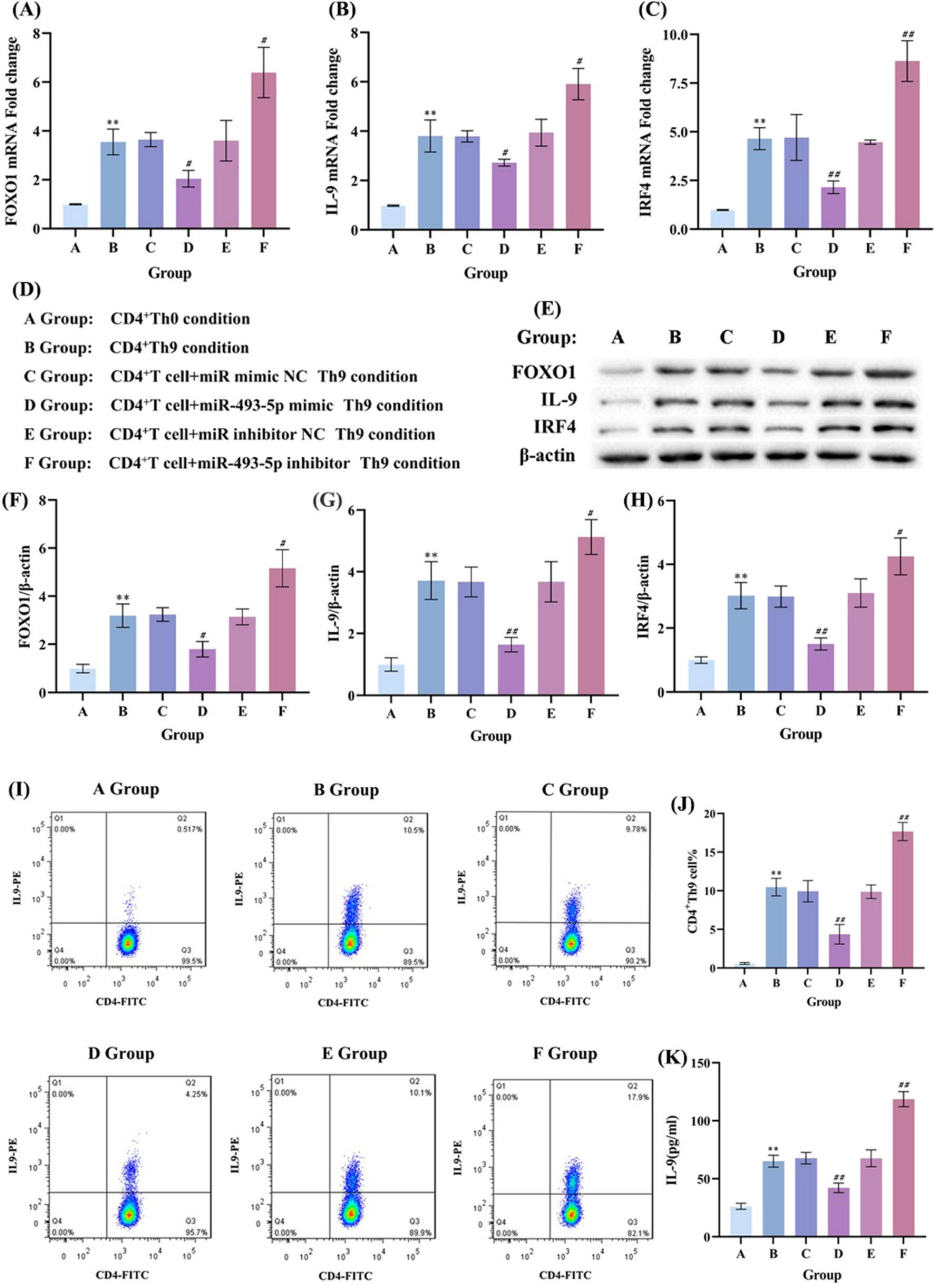
MiR-493-5p negatively regulated the differentiation of Th9 cells in vitro. The CD4^+^T cells were separated from widetype mice by density gradient centrifugation and magnetic beads. To investigate the effect of miR-493-5p on Th9 cells differentiation, both CD4^+^T cells which were transfected with miR-493-5p mimic or treated with the inhibitor and their negative control (NC) were under the condition of inducing Th9 cell differentiation. **A**–**C** The mRNA level of IL-9, IRF4 and FOXO1 was detected by RT-qPCR. The data showed that the mRNA of FOXO1, IL-9 and IRF4 were downregulated in CD4^+^T cells treated with miR-493-5p mimic and upregulated in CD4^+^T cells treated with miR-493-5p inhibitor. **D** Groups. **E**–**H** The protein production of IL-9, IRF4 and FOXO1 was detected by western blot. The data showed that protein of FOXO1, IL-9 and IRF4 were downregulated in CD4^+^T cells treated with miR-493-5p mimic and upregulated in CD4^+^T cells treated with miR-493-5p inhibitor. **I**, **J** The proportion of CD4^+^Th9 cells was analysed by flow cytometry and **K** IL-9 secretion in cell supernatant was measured by ELISA, and the data were all consistent with the proportion of Th9 cells. Data are from three experiments (mean and SD of three independent replicates).^**^P < 0.01 compared to A group, ^***#***^*P* < 0.05 compared to B group, ^***##***^*P* < 0.01 compared to B group

To investigate the effect of miR-493-5p on Th9 cells differentiation, we isolated CD4^+^T cells from the peripheral blood of widetype mice and cultured in vitro as the control group (A group). Then, the cells were transfected with miR-493-5p mimic/inhibitor under the condition of inducing Th9 cell differentiation. The overexpression / knockout efficiency of miR-493-5p mimic/inhibitor was shown in Additional file 1: Fig. S2A. The RT-qPCR (Fig. 3A–C) and Western blot (Fig. 3E–H)data showed that the mRNA and protein of FOXO1, IL-9 and IRF4 were downregulated in CD4^+^T cells treated with miR-493-5p mimic and upregulated in CD4^+^T cells treated with miR-493-5p inhibitor. Also, the Th9 cells differentiation (by flowcytometry, Fig. 3I and J) and the change trend of IL-9 level (by ELISA, Fig. 3K) was consistent with the proportion of Th9 cells. These results suggested that miR-493-5p could negatively regulate the expression of FOXO1, IL-9 and IRF4 and the differentiation of Th9 cells in vitro. The experiment repeated 3 times.

### MiR-493-5p targetingFOXO1

**Fig. 4:**
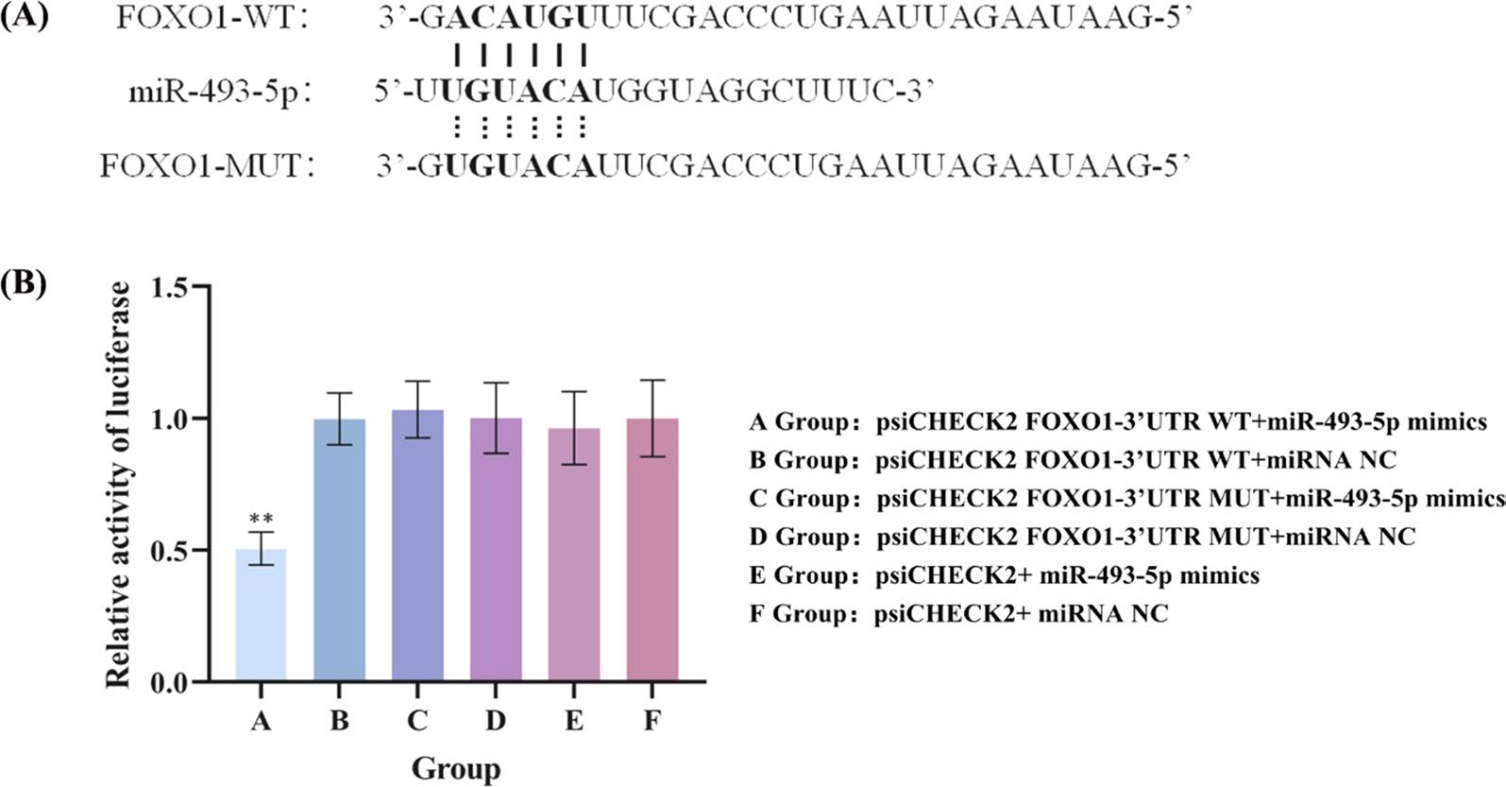
MiR-493-5p directly targets FOXO1. **A** The binding sites of miR-493-5p on the 3′UTR of FOXO1, as well as the mutant sites based on TargetScan. **B** Dual-luciferase reporter assay was launched to assess the luciferase activities of FOXO1 3′UTR WT and FOXO1 3′UTR MUT reporters in cells after miRNA NC or miR-493-5p mimic transfection.^**^P < 0.01, compared to Group F.

As the TargetScan predicted FOXO1 was a target gene of miR-493-5p, we performed the dual-luciferase reporter assay to verify the relationship between miR-493-5p and FOXO1. The FOXO1-3′UTR WT or FOXO1-3′UTR MUT seed region was amplified by PCR, and then cloned into a psiCHECK-2 vector (Promega Corporation), downstream of the Renilla luciferase gene. As Fig. 4A showed, the relative luciferase activity significantly decreased in cells co-transfected with FOXO1-3′UTR-WT and miR-493-5p mimic than cells co-transfected FOXO1-3′UTR-WT and miRNA NC. However, the relative luciferase activity didn't change in cells than co-transfected with miR-493-5p mimic and FOXO1-3’UTR MUT or with FOXO1-3′UTR MUT and miRNA NC (Fig. 4B), which suggested that miR-493-5p could directly target the 3′UTR of FOXO1 by binding with it.

**Fig. 5:**
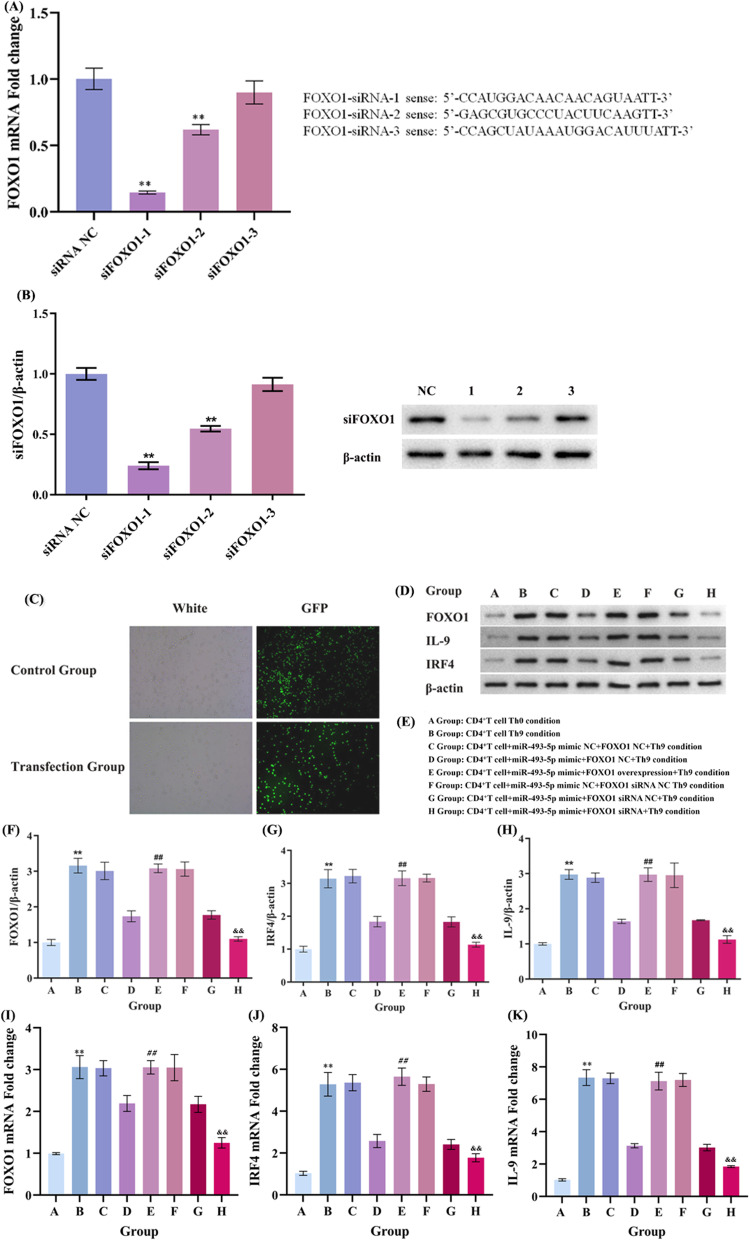
MiR-493-5p regulates Th9 cells differentiation by targeting FOXO1. To investigate whether miR-493-5p regulates Th9 cell differentiation by targeting FOXO1, the CD4^+^T cells were co-transfected with miR-493-5p mimic and FOXO1 overexpressing plasmids/interference sequences under the condition of inducing Th9 cell differentiation. **A**, **B** We prepared 3 targeted interference sequences for FOXO1, and the knockdown efficiency of FOXO1 interference sequences was detected by RT-qPCR and Western blot. The result showed that the FOXO1-1 siRNA was the best interference sequences, which was used for the following experiments. **C** The fluorescent tracker show that the FOXO1 overexpression and miR-493-5p overexpression were present in these cells. **E** The isolated Naïve CD4^+^T cells were divided into 8 groups, group. **D**, **F**–**H** Western blot: group *C*–*E* show that overexpression of FOXO1 can reverse the overexpression of miR-493-5p, and group *F*–*H* show that interference of FOXO1 can reverse the knockdown of miR-493-5p. **I**–**K** PCR: group *C*–*E* show that overexpression of FOXO1 can reverse the overexpression of miR-493-5p, and group *F*–*H* shows that interference of FOXO1 can reverse the knockdown of miR-493-5p. ^**^P < 0.01 compared to A group, ^***##***^*P* < 0.01 compared to *D* group,^***&&***^*P* < 0.01 compared to *G* group

Our experimental results show that miR-493-5p could negatively regulate the differentiation of Th9 cells in vitro, and directly binding with the 3′-UTR of FOXO1. We speculated that miR-493-5p regulated the Th9 cells differentiation by targeting FOXO1. To certificate our speculation, we prepared 3 targeted interference sequences for FOXO1 (Fig. 5A), and the knockdown efficiency of FOXO1 interference sequences was detected by RT-qPCR. Results show that the FOXO1-1 siRNA was the best interference sequences (Fig. 5A), which was used for the following experiments. Moreover, the overexpression efficiency of FOXO1 was detected by RT-qPCR and western blot (Additional file 1: Fig. S2B, C). The isolated Naïve CD4^+^T cells were divided into 8 groups, Western blot and PCR show that overexpression of FOXO1 can reverse the overexpression of miR-493-5p, and interference of FOXO1 can reverse the knockdown of miR-493-5p.

The isolated Naïve CD4^+^T cells were divided into 6 groups, and cells in group B-F were co-transfected with miR-493-5p mimic and FOXO1 overexpressing or siRNA plasmids under the condition of inducing Th9 cell differentiation. Compared with group E, the mRNA (Fig. 5B–D) and protein production (Fig. 5E, G–I) of FOXO1, IL-9 and IRF4, and IL-9 secretion (Fig. 5L) in group F significantly decreased. Meanwhile, there was no significantly difference between group C and D. The change trend of Th9 cells proportion is parallel to the mRNA and protein production of FOXO1, IL-9 and IRF4 (Fig. 5J and K). The results showed that overexpression of FOXO1 in the Th9 cells offset the inhibitory effect of miR-493-5p mimic on Th9 cells differentiation, while knockdown FOXO1, the inhibitory effect of miR-493-5p mimic was strengthened. These results indicated that miR-493-5p negatively regulated the Th9 cells differentiation by targeting FOXO1.

### MiR-493-5p’s role in allergic asthma

**Fig. 6:**
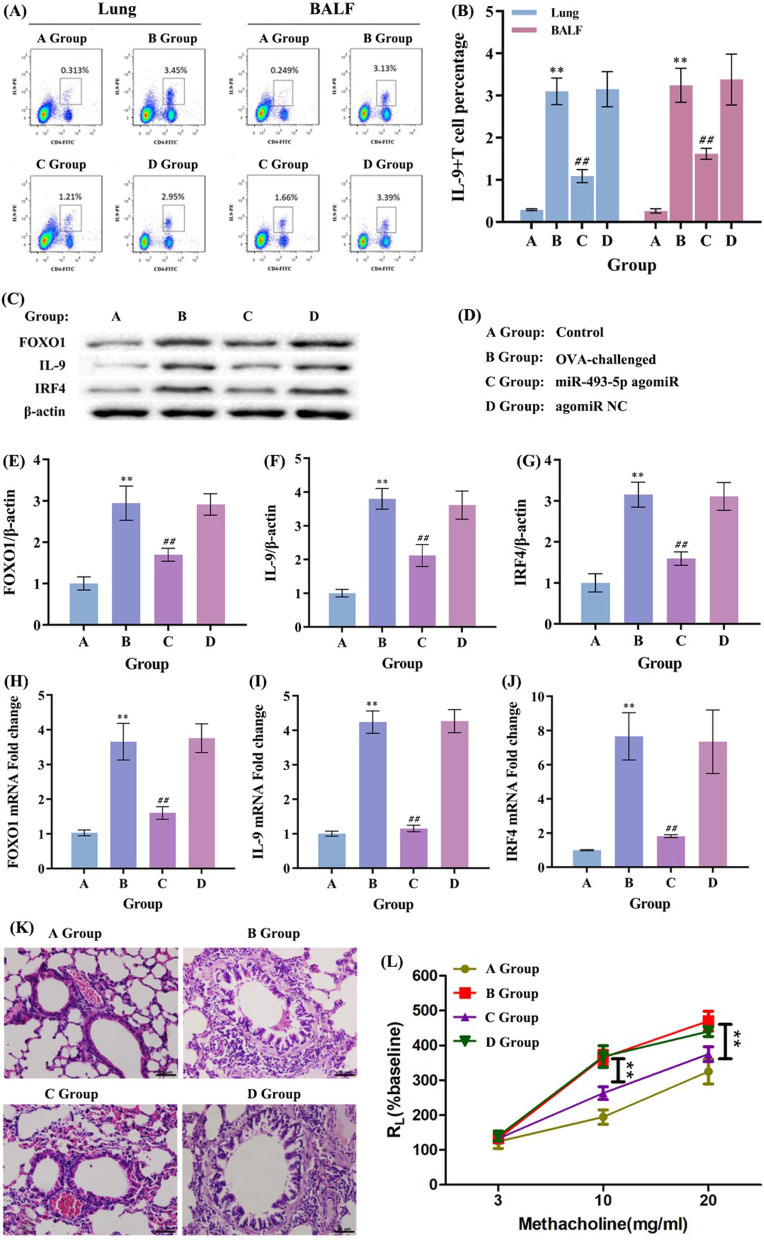
MiR-493-5p agomiR attenuates allergic airway inflammation and airway hyperreactivity. BALB/c mice were divided into 4 groups (n = 8 for each group), control, asthma, miR-493-5p agomiR and agomiR NC. MiR-493-5p agomiR or agomiR NC was dropped into the nasal cavities of the asthma model mice before the models were stimulated by OVA. **A**, **B** miR-493-5p agomiR treatment significantly reduced the proportion of Th9 cells, and the protein production of FOXO1, IL-9 and IRF4 in OVA-induced asthma mice were significantly reduced. **C** The protein production of FOXO1, IRF4 and IL-9 in lung tissue was detected by western blot. **D** Groups. **E**–**G** The protein production of FOXO1, IRF4 and IL-9 in lung tissue was analyzed by Western blot and quantified using ImageJ software. **H**–**J** The mRNA expression of IL-9, IRF4 and FOXO1 was detected by RT-qPCR. **K** The HE staining results showed a large number of inflammatory cells infiltrated around the bronchi and blood vessels, inflammatory cells and exudates increased, epithelial cells edema and lumen stenosis were observed in OVA-induced asthma mice. Compared with the OVA-induced asthma mice following with agomiR NC treatment, the inflammatory reaction dramatically attenuated in miR-493-5p agomiR treatment mice. **L** The airway hyperreactivity, miR-493-5p agomiR treatment significantly inhibited the airway hyper-responsiveness in OVA-induced asthma mice (**P < 0.01 compared to agomiR NC group). ^**^P < 0.01 compared to A group, ^**##**^*P* < 0.01 compared to B group

To investigate the role of miR-493-5p in allergic asthma, we used nasal drops of miR-493-5p agomiR or agomiR NC to OVA-immunized mice 3 days before airway challenge with OVA. 24 h after the last challenge, all the mice were anesthetized for the measurement of pulmonary mechanics, and then euthanasia to collect the lung tissue and BALF. As Fig. 6A, B shown, miR-493-5p agomiR treatment significantly reduced the proportion of Th9 cells, and the protein production of FOXO1, IL-9 and IRF4 in OVA-induced asthma mice were significantly reduced (Fig. 6C, E–G).

In addition, the HE staining results showed a large number of inflammatory cells infiltrated around the bronchi and blood vessels, inflammatory cells and exudates increased, epithelial cells edema and lumen stenosis were observed in OVA-induced asthma mice. Compared with the OVA-induced asthma mice following with agomiR NC treatment, the inflammatory reaction dramatically attenuated in miR-493-5p agomiR treatment mice (Fig. 6K). Furthermore, miR-493-5p agomiR treatment significantly inhibited the airway hyper-responsiveness in OVA-induced asthma mice (Fig. 6L). These results showed that miR-493-5p agomiR suppressed the inflammation and Th9 cell differentiation in asthmatic mice**.**

### DC-derived exosomal miR-493-5p inhibit Th9 differentiation

**Fig. 7:**
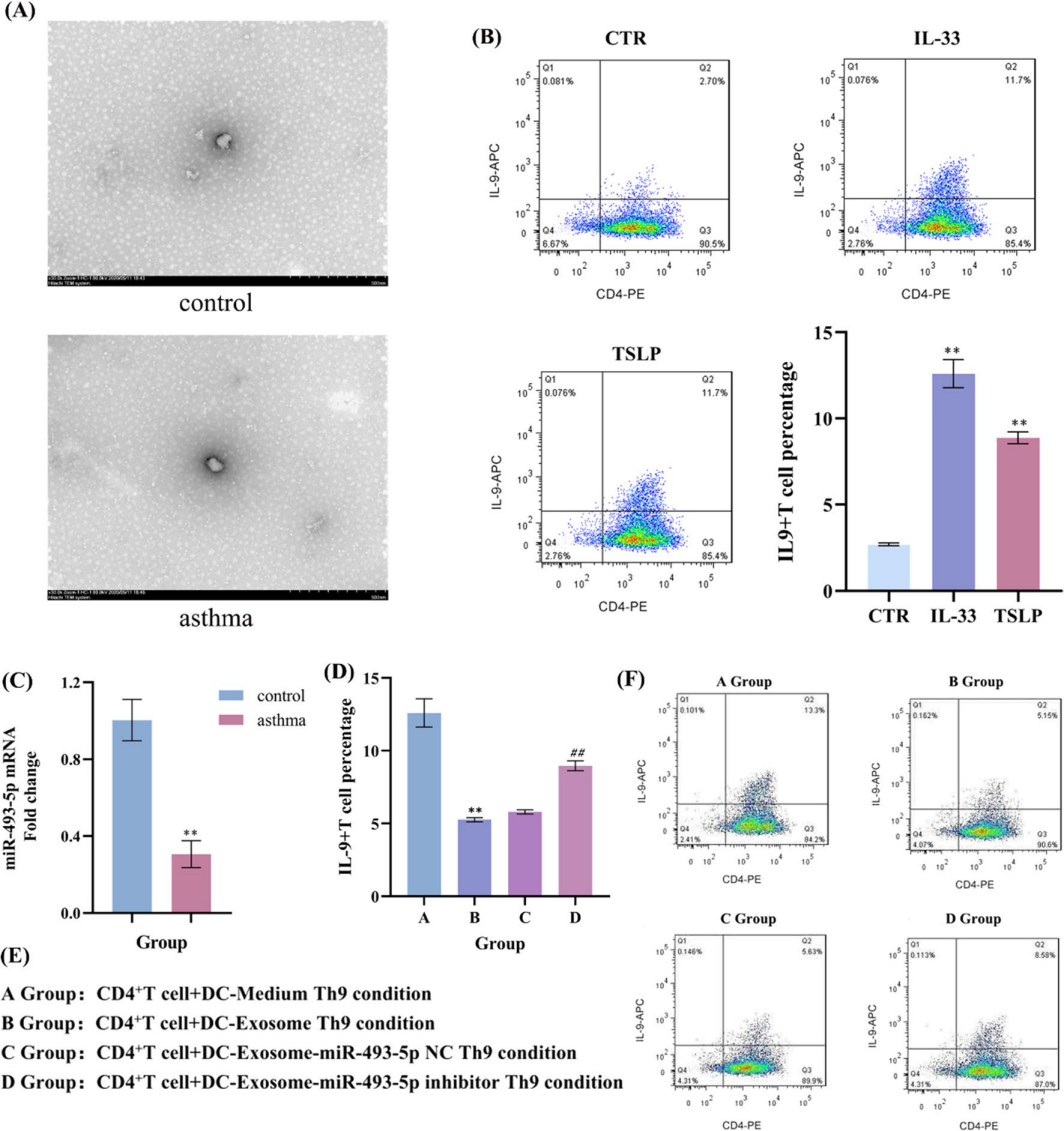
DC-derived exosomal miR-493-5p inhibit Th9 differentiation. DC-derived exosomes were incubated with CD4^+^T cells in Th9 condition, and IL-9 were detected by flow cytometry. BMDC-derived exosome-miR-493-5p NC and exosome-miR-493-5p inhibitor were constructed and incubed with naïve T cells in Th9 condition respectively, and IL-9 were detected by flow cytometry. **A** Exosomes isolated from DC. **B** The proportion of CD4^+^Th9 cells was analysed by flow cytometry. **C** The mRNA expression of miR-493-5p was detected by RT-qPCR. **D**, **F** The proportion of CD4^+^Th9 cells was analysed by flow cytometry. **E** Groups. ^**^P < 0.01 compared to control group

We incubated the naïve CD4^+^T cells with BMDC-derived exosomes in Th9 condition, and found that exosomes can promote Th9 differentiation (Fig. 7B). By isolating exosomes from peripheral blood of asthmatic mice and control mice, we found that the expression level of miR-493-5p was significantly reduced in asthmatic mice (Fig. 7C).Exosome derived from DCs inhibited Th9 cell differentiation and promoted Th9 cell differentiation after downregulation of miR-493-5p in Exosome(Fig. 7D). All the above results proved that DC-derived exosome inhibited Th9 cell differentiation through miR-493-5p.

## Conclusions and discussions

Bronchial asthma in children is a type of chronic airway inflammation caused by immune regulation disorder [17]. Th1 cells were considered to be the protective factor of bronchial asthma. Many classic pathological mechanisms of asthma attribute this to the imbalance of Th1/Th2 cells [18, 19]. However, studies have found that the imbalance theory is different from the experimental results. Blocking Th2 cytokines cannot effectively alleviate asthma, so it cannot fully explain the pathological mechanism of bronchial asthma [20]. Th9 cell is a new type of CD4^+^T cell subtype, which is named because it mainly secretes IL-9. For a long time, IL-9 has been regarded as a related factor secreted by Th2 cells. Until 2008, Th9 cells were discovered as a new type of CD4^+^T cells [21]. Although Th2 cells can also secrete IL-9, the amount of IL-9 secreted by Th2 cells is far less than that of Th9 cells. It was proved that IL-9 in peripheral blood of asthmatic children mainly came from Th9 [22]. Also, studies have further confirmed that the CD4^+^T cells producing IL-9 showed completely different Th cell subtypes of Th9: ① Compared with Th2, Th9 secretes more IL-9, while other cytokines related to Th2, such as IL-4, IL-5 and IL-13, secrete less; ② Th9 cells did not express GATA binding protein 3 (GATA-3), orphan associated receptor (ROR-γT) and forkhead box protein P3 (Foxp3), the key transcription factors of Th2, Th17 and Treg, respectively [23].

A large number of studies have shown that Th9 is involved in the pathogenesis of asthma. In human asthma, Neurath et al. have found that Th9 and IL-9 in the peripheral blood of adult asthma patients were higher than those of healthy people [24]. IL-9 protein was found in the sputum of asthmatic patients, and the expression of IL-9 increased with the aggravation of the disease [25]. A similar phenomenon was also found in asthmatic children. Jia et al. have found that IL-9 level is significantly elevated in allergic asthma children, along with the existence of antigen-specific Th9 cells, suggesting that Th9 cells may be the major source of IL-9 in children with allergic asthma [22]. In addition, animal experiments showed that not only the proportion of Th9 cells in peripheral blood and the level of IL-9 in asthma model mice were significantly higher than those in control group, but also the level of IL-9 mRNA in lung tissue was significantly increased [26]. Our study observed IL-9 level in lung tissue and BALF and the proportion of CD4^+^Th9 cells in the PBMCs of asthmatic children were significantly higher than that of non-asthmatic children, which was consistent with the existing reporters.

Different cytokines can affect the differentiation of primitive CD4^+^T cells into different subtypes. TGF-β and IL-4 can promote Th cells to differentiate into Th9 cells. According to Veldhoen’s study, in the absence of IL-6, the synergistic effect of TGF-β1 and IL-4 can promote the highly polarized primitive Th cells to differentiate into Th9 cells [27]. In another experiment, when Dardalhon studied the original CD4^+^Foxp32^−^CD62l^+^T cells from mice, he also found that under the combined effect of TGF-β1 and IL-4, the original cells differentiated into Foxp3^−^effector T cells that produced IL-9 and IL-10, namely Th9 cells [21]. Herein, we successfully induced CD4^+^T cells to differentiate into Th9 cell with TGF-β and IL-4. Studies have shown that the development of Th9 and IL-9-producing T cells requires a unique transcription factor, such as IRF4 [28], BATF3 [29] and IRF1 [4],which have been proved to be transcription factors of IL-9 gene and regulate the transcription of IL-9 gene by binding to the promoter during Th9 cell differentiation. FOXO1 can bind to IL-9 and IRF4 promoters and promote IL-9 production by T cells. Inhibition ofFOXO1 can inhibit IL-9 secretion by T cells [6]. In this study, we also found significantly upregulated IRF4, FOXO1 and IL-9 in lung tissues of asthma mice, and these mRNA and protein expression were all increased under the condition of inducing Th9 cell differentiation, which was in agreement with the result reported before.

Abnormal miRNA expression has been extensively reported and considered as a marker of human diseases. Many studies reported miRNAs were involved in the pathogenesis of asthma [30, 31]. But as a tumor suppressor gene, the expression and role of MiR-493-5p in asthma is still unknown. In this study, PBMCs were isolated from peripheral blood of asthmatic children, and high throughput sequencing was used to screen different expression of miRNAs. The informatics analysis results showed that miR-493-5p, which was related to Th cell differentiation, was downregulated in PBMCs of asthmatic children. Further studies showed that the expression of miR-493-5p was downregulated in lung tissue and BALF of asthmatic mice, and miR-493-5p could negatively regulate the differentiation of Th9 cells in vitro. Animal experiments showed that miR-493-5p agomiR attenuated allergic airway inflammation and airway hyperreactivity, and FOXO1 was identified as a direct target of miR-493-5p from a dual-luciferase reporter system. The subsequent rescue experiments proved that miR-493-5p regulating the Th9 cells differentiation by targeting FOXO1.

Many studies demonstrated that exosomes secreted by cells play an indispensable role in intercellular communication [32] by carrying biomolecules, including proteins, messenger RNAs (mRNAs), DNA, and noncoding RNAs (ncRNAs) [33]. Exosomes transferring ncRNAs (i.e. miRNAs) can also influence diverse biological processes in other cells [8]. Emerging evidence shows that exosomes or exosomal miRNAs released by asthma-associated cells, such as mast cells [34], eosinophils [35], neutrophils [36], and T lymphocytes [37], can function as mediators of intercellular information exchange, thereby contributing to AHR, airway inflammation. Exosomes from DCs activated by thymic stromal lymphopoietin (TSLP) will impact Th2 cell differentiation [38]. IL-33 activates mouse bone marrow-derived myeloid DCs to prime naïve T cells to produce IL-5 and IL-13 [39, 40], and to directly stimulate mouse naïve CD4^+^ cells to differentiate into Th2 cells producing IL-5 and IL-13. In this study, we found that TSLP and IL-13 activated DCs to release exosomes which can promote Th9 differentiation. Our PCR detection results of exosomes extracted from asthmatic mice also showed a significant drop of the miR-493-5p expression level. Therefore, DC-derived exosome can inhibit and promote Th9 cell differentiation after down-regulation of miR-493-5p.

In conclusion, this study demonstrated that miR-493-5p inhibits airway inflammation in asthma through suppressed FOXO1 expression to reduce Th9 cell differentiation, and DC-derived exosome inhibited Th9 cell differentiation through miR-493-5p, thus DC-derived exosomal miR-493-5p /FOXO1/Th9 may serve as a potential therapeutic target in the development of asthma.

## Supplementary Information

**Additional file 1: Figure S1. **mRNA changes of miR-493-5p and partial cytokines related to Th9 cell differentiation in mouse asthma model. ^**^P < 0.01, compared to control. **Figure S2**. Validation of overexpression/knockout efficiency. (A) The overexpression/knockout efficiency of miR-493-5p mimic/inhibitor. (B) The overexpression efficiency of FOXO1.

## Data Availability

All data generated and/or analyzed during this study are available from the corresponding author on reasonable request.
