# Supplementary material for: MiR-493-5p inhibits Th9 cell differentiation in allergic asthma by targeting FOXO1
Source: Respir Res. 2022 Oct 17;23:286. doi: 10.1186/s12931-022-02207-2 (PMC9578235; doi:10.1186/s12931-022-02207-2)

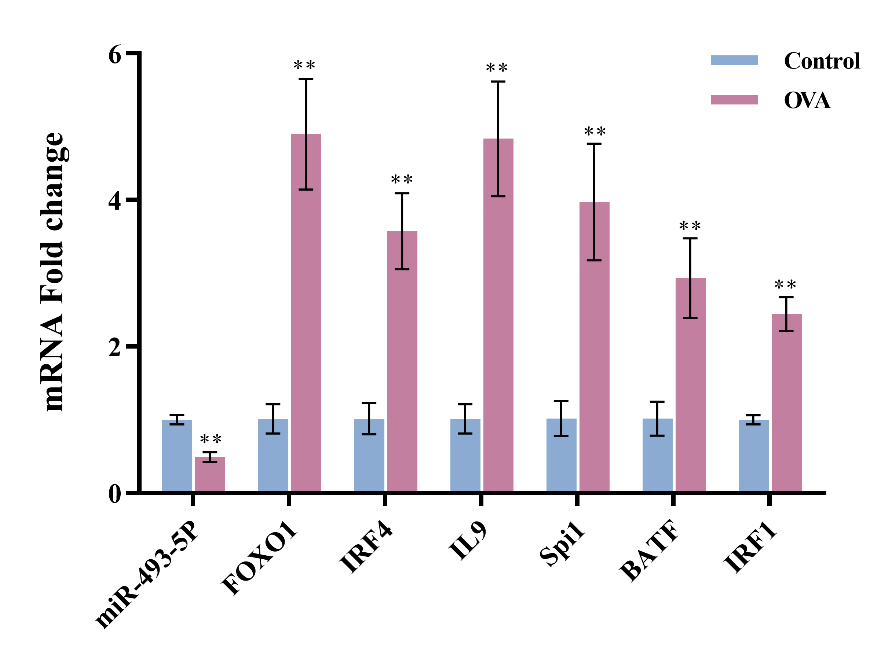
**Figure S1. mRNA changes of miR-493-5p and partial cytokines related to Th9 cell differentiation in mouse asthma model.**

**^**^***P<*0.01, compared to control.

**Figure S2.** **Validation of overexpression/knockout efficiency.**

(A) The overexpression / knockout efficiency of miR-493-5p mimic/inhibitor.

(B) The overexpression efficiency of FOXO1.


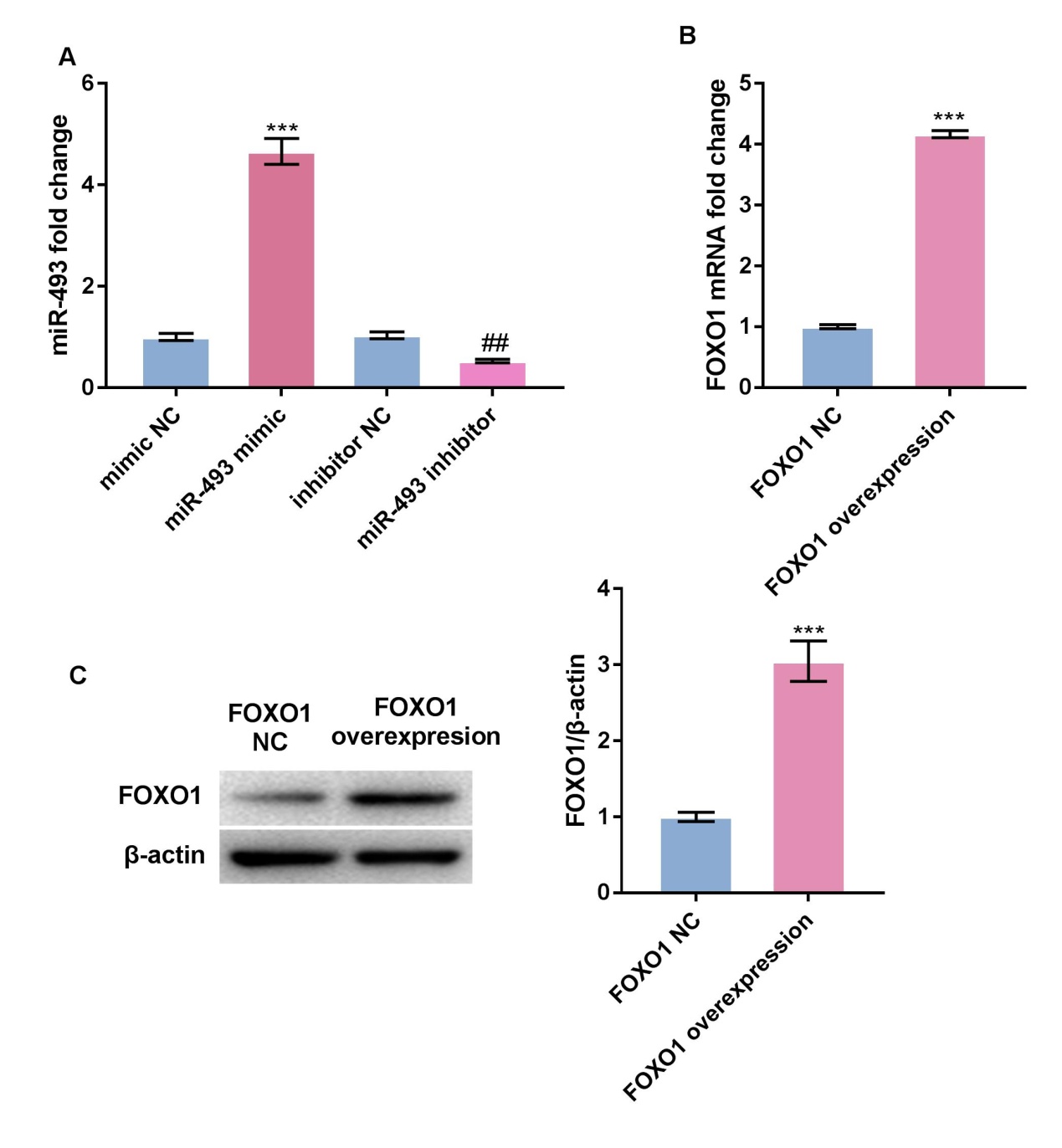

Supplement: Supplementary file 1 — Additional file 1: Figure S1. mRNA changes of miR-493-5p and partial cytokines related to Th9 cell differentiation in mouse asthma model. **P < 0.01, compared to control. Figure S2. Validation of overexpression/knockout efficiency. (A) The overexpression/knockout efficiency of miR-493-5p mimic/inhibitor. (B) The overexpression efficiency of FOXO1. [file 12931_2022_2207_MOESM1_ESM.docx]
